# Z-Ring-Associated Proteins Regulate Clustering of the Replication Terminus-Binding Protein ZapT in Caulobacter crescentus

**DOI:** 10.1128/mBio.02196-20

**Published:** 2021-01-26

**Authors:** Shogo Ozaki, Yasutaka Wakasugi, Tsutomu Katayama

**Affiliations:** a Department of Molecular Biology, Graduate School of Pharmaceutical Sciences, Kyushu University, Higashi-ku, Fukuoka, Japan; Johns Hopkins University School of Medicine; Massachusetts Institute of Technology

**Keywords:** *Caulobacter crescentus*, DNA replication, cell division, chromosome organization, subcellular localization

## Abstract

Regulated organization of the chromosome is essential for faithful propagation of genetic information. In the model bacterium Caulobacter crescentus, the replication terminus of the chromosome is spatially arranged in close proximity to the cytokinetic Z-ring during the cell cycle. Although the Z-ring-associated proteins ZapA and ZauP interact with the terminus recognition protein ZapT, the molecular functions of the complex that physically links the terminus and the Z-ring remain obscure. In this study, we found that the physical linkage helps to organize the terminus DNA into a clustered structure. Neither ZapA nor ZauP was required for ZapT binding to the terminus DNA, but clustering of the ZapT-DNA complexes over the Z-ring was severely compromised in cells lacking ZapA or ZauP. Biochemical characterization revealed that ZapT, ZauP, and ZapA interacted directly to form a highly ordered ternary complex. Moreover, multiple ZapT molecules were sequestered by each ZauP oligomer. Investigation of the functional structure of ZapT revealed that the C terminus of ZapT specifically interacted with ZauP and was essential for timely positioning of the Z-ring *in vivo*. Based on these findings, we propose that ZauP-dependent oligomerization of ZapT-DNA complexes plays a distinct role in organizing the replication terminus and the Z-ring. The C termini of ZapT homologs share similar chemical properties, implying a common mechanism for the physical linkage between the terminus and the Z-ring in bacteria.

## Abstract (precis)

Rapidly growing bacteria experience dynamic changes in chromosome architecture during chromosome replication and segregation, reflecting the importance of mechanisms that organize the chromosome globally and locally within a cell to maintain faithful transmission of genetic material across generations. During cell division in the model bacterium Caulobacter crescentus, the replication terminus of the chromosome is physically linked to the cytokinetic Z-ring at midcell.

## INTRODUCTION

Sequestration of individual chromosomal loci to specific subcellular positions plays a fundamental role in cell proliferation and differentiation. In vertebrates, chromosomes are compactly organized in the nucleus, forming multiscale structural units. The positioning of these units relative to the nuclear periphery impacts the transcriptional activity of specific genes and, thus, they serve as *cis*-acting elements to control chromosome replication and cell division (1–3). In bacteria, accurate positioning of chromosomes enables cells to coordinate chromosome replication and segregation with cell division to ensure faithful transmission of genetic material over generations (4–6). To achieve these goals, diverse DNA-binding proteins in complex with distinct chromosomal loci are localized at specific times and subcellular sites. However, the underlying localization mechanisms and functional structures of the dedicated DNA-binding proteins remain incompletely defined.

**FIG 1:**
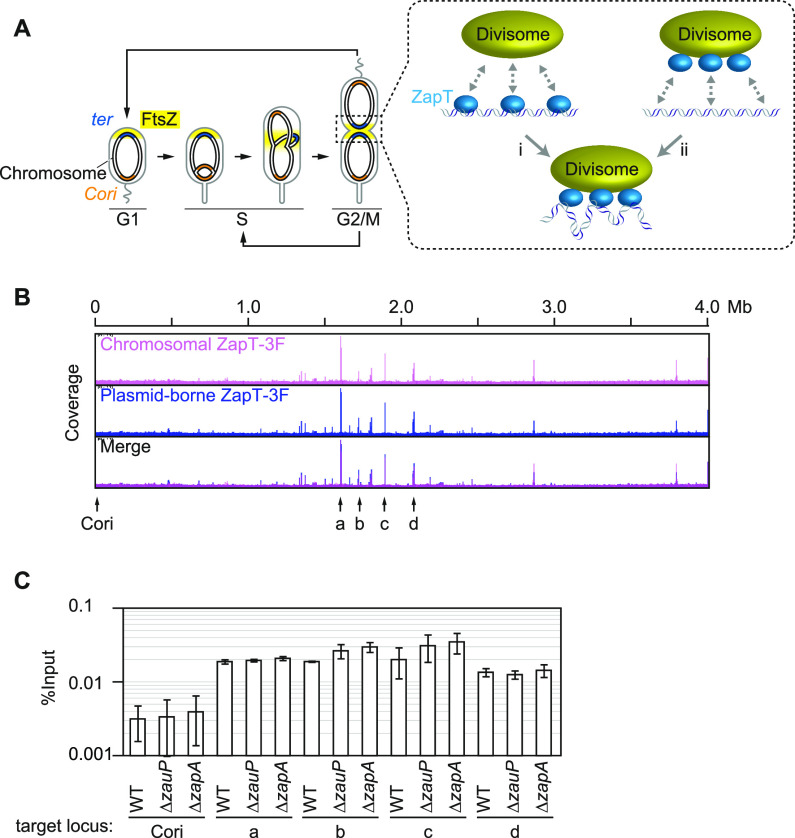
ZauP and ZapA are dispensable for the interaction of ZapT with the terminus DNA. (A) Cell cycle of C. crescentus. Localization of the origin of replication (*Cori*, orange), terminus (*ter*, blue), and FtsZ (yellow) at distinct cell cycle stages (G_1_, S, and G_2_/M) is shown schematically. Two possible modes of DNA-ZapT-divisome interaction are also depicted: (i) the preformed ZapT-DNA complexes interact with the divisome; (ii) the preformed ZapT-divisome complex interacts with DNA. (B) ChIP sequencing (ChIP-seq) analysis for ZapT. A standard ChIP-seq protocol was applied to cells expressing ZapT-3F from the native locus (SHQ10; pink) or a low-copy-number plasmid (SHQ176; blue). Coverage was plotted against the NA1000 genomic position. Arrows (*Cori*, a to d) indicate the loci analyzed in panel C. (C) DNA-binding activity of ZapT in the absence of ZapA or ZauP. The pQF::*zapT* plasmid was introduced into the Δ*zapT* mutant or its derivative strains lacking *zapA* or *zauP*. The cells were grown exponentially, followed by chromatin immunoprecipitation with an anti-FLAG antibody. Recovery of the indicated genomic positions was analyzed by qPCR and plotted as percent input. Mean values and standard deviations were obtained from two biological replicates. Locus-specific qPCR primers were used: 11/12 for Cori, 694/695 for a, 683/684 for b, 9/10 for c, and 687/688 for d.

In the model organism Caulobacter crescentus, an aquatic alphaproteobacterium with asymmetric division, subcellular positioning of individual chromosomal loci changes dynamically in a manner that is coordinated with the cell cycle (7). In this bacterium, cell cycle progression produces two genetically identical but physiologically distinct progeny cells (Fig. 1A) (8–12). The stalked progeny is sessile and can initiate chromosome replication and cell division (entry into S phase), whereas the motile swarmer progeny cannot undergo replication initiation and consequently experiences an extended nonproliferating period, termed the G_1_ phase. In the G_1_ phase, the origin of chromosome replication is located at the flagellated cell pole, the site of future stalk morphogenesis. During the G_1_-to-S transition, the cell undergoes flagellar ejection and stalk morphogenesis and concomitantly regains the ability to initiate replication at the origin. Subsequently, one of the replicated origins translocates dynamically from the incipient stalked cell pole to the opposite cell pole. This active translocation of the origin is mediated by the essential ParA-ParB-*parS* system (13–16). The centromere-like *parS* sequence proximal to the origin is specifically recognized by the ParB DNA-binding protein (17–19). The resultant ParB-*parS* complexes associate with the ParA ATPase, which generates a driving force for translocation of the *parS*-proximal DNA region including the origin (16, 20). Finally, the pole-localizing protein PopZ captures the segregated ParB complexes at cell poles, ensuring bipolar sequestration of the sister origins (21–23).

The replication terminus of C. crescentus, which is located on the opposite side of the chromosome from the origin of replication, also undergoes dynamic changes in subcellular position during S phase (7, 24) (Fig. 1A). The terminus region is initially sequestered at the cell pole opposite the origin-tethering cell pole in G_1_ phase. Upon S phase entry, the terminus region relocates to the midcell region, where cell division takes place. This central positioning of the terminus DNA persists as the cell cycle progresses, and each of the sister-replicated terminus DNAs is deposited to the new cell poles of the individual daughter cells following division. Notably, this dynamic behavior of the terminus DNA coincides with the subcellular positioning of the cytokinetic Z-ring, a bundle of filamentous polymers formed by the essential GTPase FtsZ (24). The midcellular localization of the Z-ring at S phase initiates cell division and provides a scaffold for the divisome, an assembly of protein complexes involved in this process (15, 25–28).

To date, we know of two different systems that account for the physical linkage between the Z-ring and the terminus DNA: the MatP-ZapB-ZapA_EC_ system in the gammaproteobacterium Escherichia coli and the ZapT-ZauP-ZapA system in C. crescentus. MatP, a terminus-specific binding protein, forms a dimer that specifically recognizes the 13-mer *matS* sites that are distributed within an 800-kb stretch around the terminus region (29–32). Structural and biochemical studies revealed that the C-terminal coiled-coils of DNA-bound MatP dimers interact to form a tetramer, allowing DNA bridging between distal *matS* sites (33). The resultant MatP-mediated DNA clustering organizes the replication terminus DNA region into a compacted DNA called the Ter macrodomain (30, 31, 34). Meanwhile, MatP binds directly to the coiled-coil ZapB protein (35, 36). Recombinant ZapB proteins exist as dimers in the crystal form and can form polymers in solution (35). Although ZapB exhibits no direct interaction with E. coli FtsZ_EC_, it physically associates with the E. coli ZapA tetramer (ZapA_EC_) (37). ZapA_EC_ consists of a long C-terminal helix and an N-terminal globular domain containing several charged residues that are important for the direct interaction with FtsZ_EC_ (38, 39). Collectively, the sequential interaction of *matS*, MatP, ZapB, ZapA_EC_, and FtsZ_EC_ is thought to physically link the terminus DNA to the Z-ring. Fluorescence microscopy revealed that the MatP-mCherry fusions form a discrete focus independently of ZapA_EC_ or ZapB (29), implying that the preassembled MatP-*matS* cluster, in association with the divisome, plays a predominant role in establishing the physical linkage.

C. crescentus uses the ZapT-ZauP-ZapA system instead of the MatP-ZapB-ZapA_EC_ system. Despite the low degree of conservation of MatP and ZapB, ZapT and ZauP homologs are widely conserved among Gram-negative bacteria (24, 40). In this system, the MerR DNA-binding protein ZapT plays a key role in recognition of the terminus DNA (24). Members of the MerR family contain similar N-terminal DNA-binding domains, central dimerization domains, and dissimilar C-terminal domains that interact with specific effectors, such as metal ions, chemicals, and oligopeptides (41, 42) (see Fig. S1 in the supplemental material). Although ZapT and MatP are not related at the sequence level, ZapT preferentially binds to *Caulobacter* genome positions ranging from 1.3 to 2.2 MB, corresponding to the chromosome terminus and its flanking regions. Moreover, ZapT directly or indirectly interacts with C. crescentus ZapA and ZauP *in vivo*. ZapA binds directly to FtsZ, whereas ZauP is a functional ZapB homolog with affinity for ZapA but not FtsZ. Therefore, a functional complex containing ZapT, ZapA, and ZauP is thought to mediate the physical interaction between the terminus and Z-ring in C. crescentus. However, the molecular functions underlying the ZapT-mediated interaction between the terminus DNA and divisome remain to be fully elucidated.

10.1128/mBio.02196-20.1 FIG S1 Sequence comparison. The secondary structure of ZapT, predicted using HHpred (https://toolkit.tuebingen.mpg.de/tools/hhpred), is shown along with that of MerR (PDB entry 5CRL). Sequence alignment of the C terminus of ZapT homologs from representative alpha-, beta-, and gammaproteobacterial species are shown. *Atu*, Agrobacterium tumefaciens; *Bhe*, Bartonella henselae; *Bja*, Bradyrhizobium japonicum; *Bru*, Brucella abortus; *Mlo*, Mesorhizobium loti; *Sme*, Sinorhizobium meliloti; *Wpi*, Wolbachia pipientis; *Pae*, Pseudomonas aeruginosa; *Prh*, Paraburkholderia rhizoxinica. Download FIG S1, EPS file, 1.3 MB. Copyright © 2021 Ozaki et al. 2021 Ozaki et al. This content is distributed under the terms of the Creative Commons Attribution 4.0 International license.

Here, we provide evidence that clustering of DNA-bound ZapT is stimulated by its association with the divisome. We demonstrate that recombinant ZapT, ZauP, and ZapA proteins interact sequentially, presumably in that order, to form a ternary complex. Moreover, we show that multiple ZapT molecules are recruited by each ZauP oligomer *in vitro*. Consistent with these observations, the subcellular localization of ZapT strictly depends on ZauP and ZapA. Finally, mutant analyses revealed that the C-terminal domain of ZapT is functionally specialized to directly interact with ZauP. Because the hydrophobic properties of the C terminus are shared among ZapT homologs, our findings suggest that the mechanism of terminus sequestration to the Z-ring is conserved. Moreover, the ZauP-dependent clustering of ZapT-DNA complexes provides insight into the role of the divisome in chromosome organization.

## RESULTS

### ZapA and ZauP are required for ZapT localization but dispensable for the interaction between ZapT and terminus DNA.

ZapT-binding sites are distributed preferentially at genome positions ranging from 1.3 to 2.2 MB, which correspond to the chromosome terminus and its flanking regions (24). Because the subcellular localization of ZapT coincides with that of the terminus and the divisome, we assumed that DNA-bound ZapT molecules interact with each other to form a nucleoprotein complex to which the divisome is recruited (Fig. 1A). Alternatively, multiple ZapT molecules could form a proteinaceous complex with the divisome to which the chromosomal ZapT-binding sites are recruited (Fig. 1A). To distinguish between these models, we assessed the dependence of the ZapT-DNA interaction on the divisome components ZapA and ZauP, both of which form a complex with ZapT *in vivo*. To investigate the DNA-binding activity of ZapT *in vivo*, we performed chromatin immunoprecipitation (ChIP) assays using strains expressing a C-terminally 3×FLAG-tagged ZapT (ZapT-3F) from a cumate-dependent promoter on a low-copy-number plasmid (pQF::*zapT*). Deep sequencing revealed that the distribution pattern of plasmid-borne ZapT-3F was indistinguishable from that of chromosomally expressed ZapT-3F (Fig. 1B). Moreover, binding of ZapT-3F to terminus-proximal regions such as the CCNA_01498, CCNA_01600, CCNA_01763, and CCNA_01936 loci (Fig. 1B, a, b, c, and d, respectively) was confirmed by quantitative PCR (ChIP-qPCR) (Fig. 1C). The origin region (Cori) was used as a negative control. Next, we performed a similar ChIP-qPCR analysis in the Δ*zapA* and Δ*zauP* backgrounds. The profiles of DNA recovery in these mutants were comparable with those in the wild-type background (Fig. 1C). Therefore, ZapA and ZauP are dispensable for the DNA-binding activity of ZapT, arguing against the idea that formation of ZapT complexes with ZapA and ZauP is a prerequisite for the ZapT-terminus DNA interaction.

**FIG 2:**
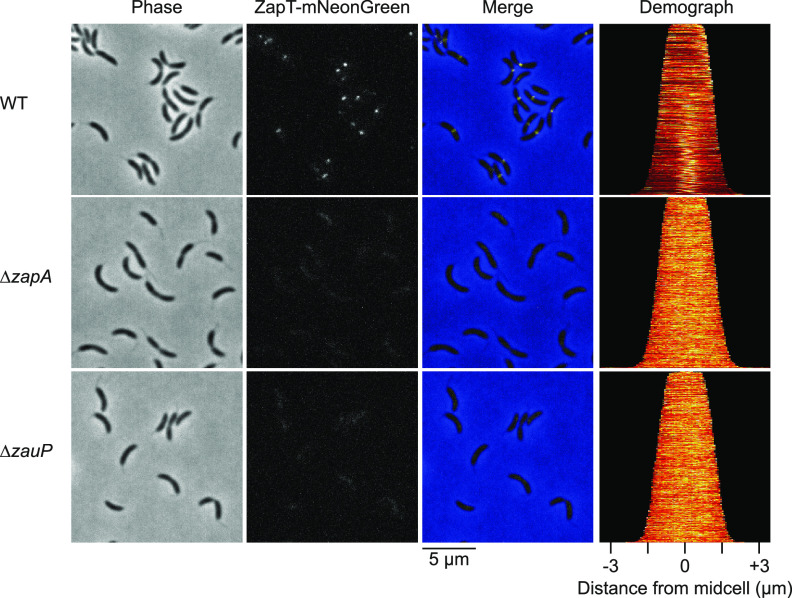
ZauP and ZapA are required for subcellular localization of ZapT. Localization of ZapT-mNeonGreen in the absence of ZapA or ZauP. SHQ143 (wild type; WT), SHQ153 (Δ*zapA*), and SHQ154 (Δ*zauP*) strains expressing ZapT-mNeonGreen from the native locus were grown exponentially in PYE medium, followed by analysis by fluorescence microscopy. Representative phase-contrast and fluorescence microscopy images are shown. Demographs were generated using Oufti software (63).

Next, we assessed the localization dependence of ZapT on *zapA* and *zauP*. To visualize the localization of ZapT, we used a strain expressing a C-terminal mNeonGreen fusion of ZapT (ZapT-mNeonGreen) from the native locus (24). We reported previously that in the wild-type strain, the localization pattern of ZapT-mNeonGreen parallels that of the Z-ring, i.e., the cells form a single discrete focus of ZapT-mNeonGreen at one cell polar region in shorter cells and at the midcell region in larger cells (24). Moreover, colocalization analysis revealed that a ZapT-mNeonGreen focus coincides with ZapA and the terminus in the same wild-type cell. Consistent with these observations, nearly all wild-type cells formed a discrete focus of ZapT-mNeonGreen at the midcell region or in one cell polar region (Fig. 2). In contrast, in cells lacking ZapA or ZauP, ZapT-mNeonGreen was completely dispersed (Fig. 2). These observations were not due to the instability of ZapT-mNeonGreen, as we obtained similar results with wild-type, Δ*zapA*, and Δ*zauP* strains transformed with a pQF::*zapT* derivative expressing ZapT-mNeonGreen instead of ZapT-3F (Fig. S2A). Western blotting demonstrated that the levels of ZapT-mNeonGreen in these mutants were indistinguishable from those in the wild type (Fig. S2B). Therefore, ZapA and ZauP are required for proper localization of ZapT. Together, these results favor a model in which ZapT-DNA complexes are recruited to the divisome through interactions with ZapA and ZauP.

10.1128/mBio.02196-20.2 FIG S2 Subcellular localization of ZapT in cells lacking ZauP or ZapA. (A) Localization of ZapT-mNeonGreen in the absence of ZapA or ZauP. The pQF::*zapT-mNeonGreen* plasmid was introduced into the wild-type, Δ*zapA*, or Δ*zauP* strain. Cells were grown exponentially in PYE medium and subjected to fluorescence microscopy. Representative DIC and fluorescence microscopy images are shown. Demographic representations were generated using Oufti software. (B) Expression of ZapT-mNeonGreen in each background was analyzed using Western blotting with an anti-mNeonGreen antibody (1:1,000) (α-mNeonGreen). The gel after protein transfer to the membrane was stained using Coomassie brilliant blue and shown as a loading control (Coomassie). Download FIG S2, TIF file, 2.4 MB. Copyright © 2021 Ozaki et al. 2021 Ozaki et al. This content is distributed under the terms of the Creative Commons Attribution 4.0 International license.

### ZapT directly binds to ZauP.

**FIG 3:**
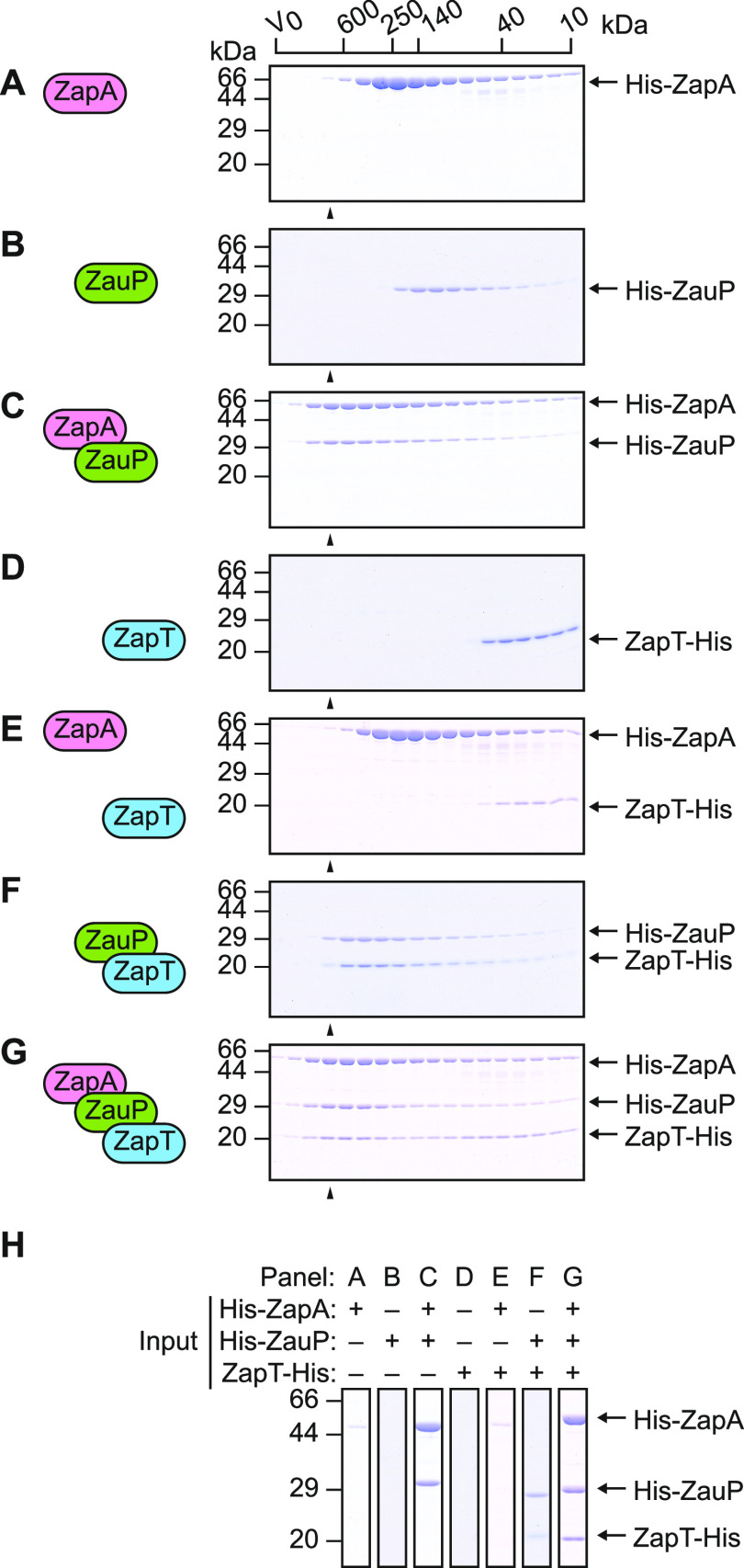
ZapT dimers bind to ZauP directly. Size exclusion chromatography is shown. His-ZapA (2.7 nmol as monomer) (A), His-ZauP (3.8 nmol as monomer) (B), His-ZapA and His-ZauP (4.0 nmol each as monomers) (C), ZapT-His (12 nmol as monomer) (D), His-ZapA and ZapT-His (5.4 nmol each as monomers) (E), His-ZauP and ZapT-His (7.2 nmol each as monomers) (F), and His-ZapA, His-ZauP, and ZapT-His (3.5 nmol each as monomers) (G) were separated using a Superdex 200 column PC3.2/30, and elution fractions (1.0 to 2.2 ml) were analyzed by SDS–15% PAGE and Coomassie brilliant blue staining. The elution positions of the molecular weight marker proteins and void volume (Vo) are indicated. (H) To compare fractions corresponding to an average molecular weight of ∼600 kDa, cropped images of lane 4 (indicated by arrowheads) from panels A to G are collectively shown.

To map interactions between ZapT, ZapA, and ZauP, we performed size exclusion chromatography on the purified recombinant proteins: ZapT with a C-terminal hexahistidine tag (ZapT-His; 21 kDa), ZapA with an N-terminal hexahistidine-MBP tag (His-ZapA; 56 kDa), and ZauP with an N-terminal hexahistidine-SUMO tag (His-ZauP; 26 kDa). First, we determined the oligomeric state of His-ZapA and His-ZauP using a Superdex 200 column (Fig. 3). Previously, ZapA homologs from P. aeruginosa and E. coli were reported to form homotetramers (38, 43). Consistent with those studies, our His-ZapA eluted at a position corresponding to the average molecular weight of a His-ZapA tetramer (220 kDa) (Fig. 3A). Likewise, we found that His-ZauP formed multimers with the average molecular weight of a His-ZauP hexamer (150 kDa) (Fig. 3B). Moreover, when mixed together, His-ZapA and His-ZauP coeluted earlier than either protein alone (Fig. 3A to C). Thus, His-ZapA and His-ZauP retain the ability to form heteromultimers *in vitro*.

Next, we used a Superdex 200 column to analyze an oligomeric state of ZapT-His. ZapT shares structural homology with transcriptional regulators of the MerR family (E value of 1.0 × 10^−16^), whose members typically form stable dimers *in vitro*. In our setup, ZapT-His eluted broadly near a position corresponding to dimeric ZapT-His (43 kDa) (Fig. 3D). To better resolve the oligomeric state, we analyzed ZapT-His using a Superdex 75 column with a separation range between 3 and 70 kDa. We detected a single elution peak of ZapT-His at a position corresponding to dimeric ZapT-His (Fig. S3). These observations argue that, as with other MerR family proteins, ZapT preferentially forms a dimer in solution.

10.1128/mBio.02196-20.3 FIG S3 Dimerization of ZapT and ZapTΔC. Size exclusion chromatography is shown. ZapT-His (A) and ZapTΔC-His (B) (60 nmol) were analyzed on a Superdex 75 column (column volume, 24 ml). The elution positions of molecular weight marker proteins are indicated above the chromatograms (UV absorbance at 280 nm). Peak fractions were analyzed by SDS–15% PAGE and Coomassie brilliant blue staining. Download FIG S3, EPS file, 2.6 MB. Copyright © 2021 Ozaki et al. 2021 Ozaki et al. This content is distributed under the terms of the Creative Commons Attribution 4.0 International license.

To probe for a direct interaction between ZapT and ZapA or ZauP, we analyzed a mixture of the two proteins using a Superdex 200 column. The chromatograms of ZapT-His and His-ZapA were unaffected by mixing the proteins together (Fig. 3A, D, and E). In contrast, in a mixture of ZapT-His and His-ZauP, the proteins were coeluted earlier than either protein alone (Fig. 3A, B, and F). This strongly argues that ZapT directly interacts with ZauP but not ZapA. The average molecular weight of the heterooligomer was 310 kDa, consistent with the idea that three or four ZapT-His dimers (130 to 170 kDa) bind to each His-ZauP hexamer (150 kDa).

Next, we analyzed a mixture of ZapT-His, His-ZapA, and His-ZauP to determine whether ZapT forms a ternary complex with ZauP and ZapA. Previously, we showed that ZapA, ZauP, and ZapT reside in the same protein complex *in vivo* (24). Consistent with this, when mixed together, ZapT-His coeluted with His-ZapA and His-ZauP at a position corresponding to an average molecular weight of >600 kDa (Fig. 3G and H), which is distinguishable from ZapT-ZauP complexes (Fig. 3F and H). Thus, ZapT sustains the ability to form a ternary structure with ZapA and ZauP.

### The C-terminal sensor domain of ZapT is required for binding to ZauP.

**FIG 4:**
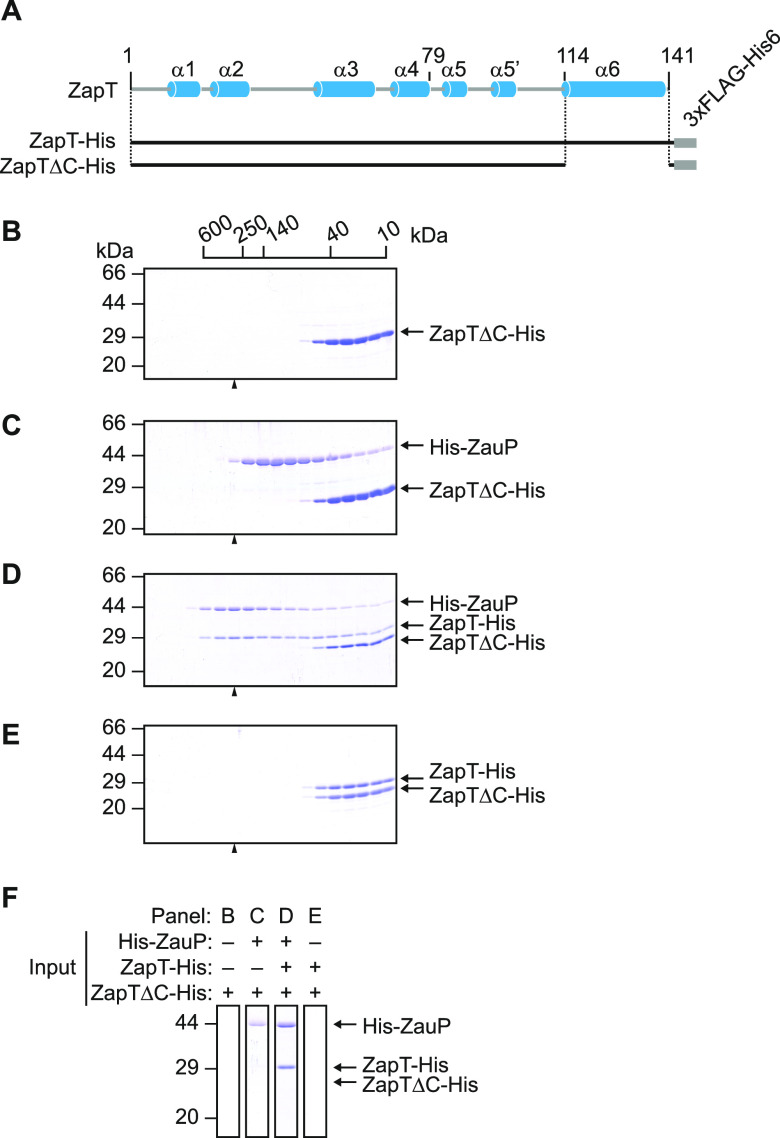
C-terminal sensor domain of ZapT is required for interaction with ZauP. (A) A C-terminally truncated variant (ZapTΔC-His) was designed based on the predicted secondary structure of ZapT shown in Fig. S1. (B to F) Size exclusion chromatography. ZapTΔC-His (15 nmol as a monomer) (B), His-ZauP and ZapTΔC-His (9.5 nmol each as monomers) (C), ZapT-His and ZapTΔC-His (10 nmol each as monomers) (D), and His-ZauP, ZapT-His, and ZapTΔC-His (6.0 nmol each as monomers) (E) were analyzed as described in the legend for Fig. 3. (F) To compare fractions corresponding to an average molecular weight of ∼250 kDa, cropped images of lane 7 (indicated by arrowheads) from panels B to E are collectively shown.

The direct interaction between ZapT and ZauP motivated us to identify the functional domain of ZapT involved in ZauP binding. A typical MerR family protein consists of three functional domains (41, 42, 44) (Fig. 4A and Fig. S1): the N-terminal DNA-binding domain, the central dimerization domain, and the C-terminal sensor domain. Because most, if not all, members of this family interact with a specific effector, such as metal ions, chemicals, or oligopeptides, through their C-terminal sensor domains, we hypothesized that the C terminus of ZapT is functionally specialized to interact with ZauP.

To determine whether the C terminus of ZapT is required for ZauP binding, we analyzed a truncated variant of ZapT-His that lacks the C-terminal sensor domain (ZapTΔC-His) using gel filtration (Fig. 4A). First, we injected the ZapTΔC-His protein into a Superdex 200 column to determine whether this variant retains dimerization activity. As shown in Fig. 4B, elution of ZapTΔC-His peaked at a position corresponding to a ZapTΔC-His dimer (29 kDa). Consistent results were obtained using Superdex 75 column chromatography (Fig. S3). Thus, the C-terminal sensor domain of ZapT is dispensable for dimerization, a finding consistent with the fact that MerR family proteins self-dimerize through the central dimerization domain. Next, we applied a mixture of ZapTΔC-His and His-ZauP to a Superdex 200 column (Fig. 4C to E). The chromatograms of ZapTΔC-His and His-ZauP were unaffected by mixing the proteins together (Fig. 4C), indicating that ZapTΔC-His impairs the ZauP binding activity. To consolidate this, we performed a similar experiment in the presence of wild-type ZapT-His as an internal control. Gel filtration of the mixture revealed that wild-type ZapT-His, but not ZapTΔC-His, coeluted with ZauP (Fig. 4D and F). Wild-type ZapT-His did not interact with ZapTΔC-His (Fig. 4E and F). Taken together, these findings strongly argue that the C-terminal domain of ZapT is crucial for interaction with ZauP.

### ZauP sequesters multiple ZapT dimers.

**FIG 5:**
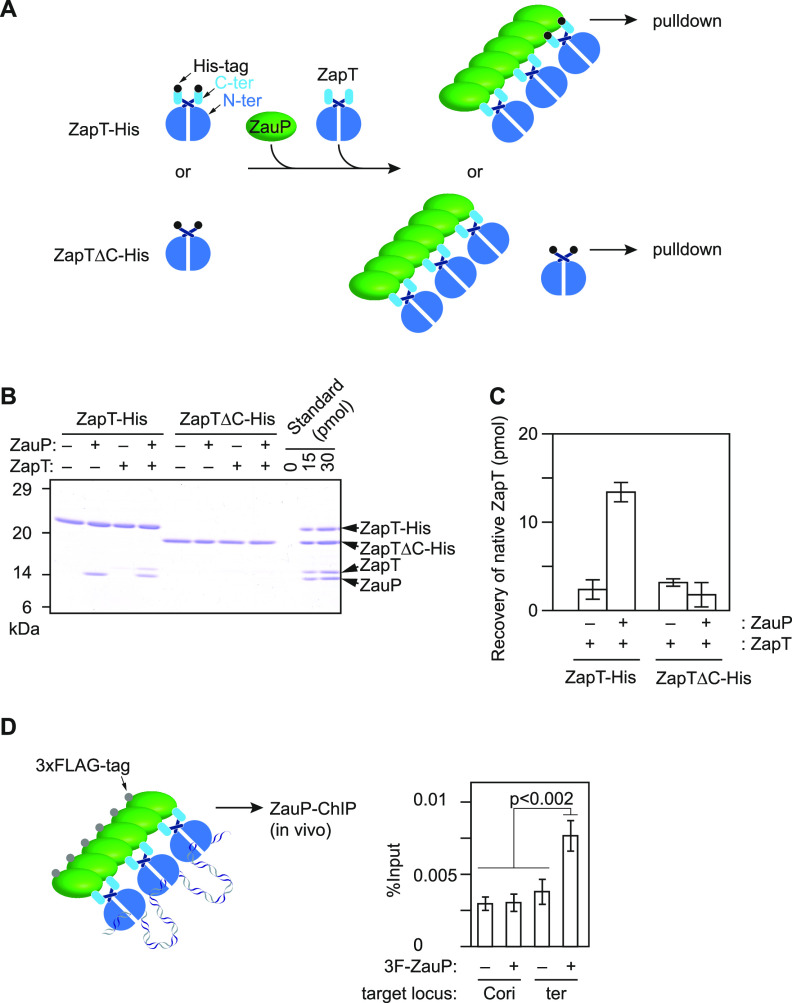
ZauP binds multiple ZapT dimers. (A) Schematic of the pulldown assay. ZapT is depicted as a blue oval with an extension (light blue) that corresponds to its C terminus. His tag (black) and ZauP (green) are indicated. (B and C) ZapT-His or ZapTΔC-His (200 pmol as monomer) was incubated in the presence (+) or absence (–) of ZauP (600 pmol as monomer) and native ZapT (300 pmol as monomer), followed by pulldown using Ni-conjugated Sepharose beads. After washing, materials retained on the beads were analyzed using SDS–15% PAGE and Coomassie brilliant blue staining. For the protein standard, the indicated amounts of purified proteins were loaded on the last two lanes of the same gel, which is used to draw a standard curve to deduce recovery of native ZapT. (C) Mean values and standard deviations obtained from two independent experiments were plotted. (D) ChIP-qPCR assay for 3F-ZauP. SHQ247 (3F-ZauP) and NA1000 were grown exponentially and cross-linked in 3.6% formaldehyde, followed by chromatin immunoprecipitation with an anti-FLAG antibody. Recovery of Cori (primers 11/12) and the terminus (position c; primers 9/10) was shown as in Fig. 1C. The *P* value was calculated using Student's *t* test.

To validate our model in which oligomeric ZauP sequesters multiple ZapT dimers, we carried out a pulldown assay using ZapT-His and intact ZapT and ZauP proteins (Fig. 5A). When ZapT-His was incubated with ZauP, we observed coelution of the two proteins (Fig. 5B). In contrast, when ZapT-His was mixed with ZapT instead of ZauP, only ZapT-His was recovered in an elution fraction (Fig. 5B and C). These observations are fully consistent with the results for the gel filtration assay (Fig. 3 and 4). Notably, when ZapT-His, ZapT, and ZauP were coincubated, we observed the specific recovery of ZapT together with His-ZapT and ZauP (Fig. 5B and C), indicating that His-ZapT, ZauP, and ZapT coexist in the same complex. Moreover, when we performed a similar analysis using His-ZapTΔC instead of His-ZapT, neither ZauP nor ZapT was coeluted with His-ZapTΔC. Thus, the C terminus of ZapT is required for formation of higher-ordered ZapT multimers on ZauP.

To further examine if the terminus-bound ZapT proteins are sequestered by ZauP *in vivo*, we carried out ChIP-qPCR experiments using a strain expressing an N-terminally 3×FLAG-tagged ZauP (3F-ZauP). When cells were cross-linked in 1% formaldehyde as with the ZapT-ChIP assay, neither the terminus nor Cori was recovered (data not shown), which is consistent with the idea that ZauP *per se* has no affinity for DNA (40). We reasoned that 1% formaldehyde cross-linking was inefficient to pull down DNA molecules that bound indirectly to ZauP. Therefore, we adopted 3.6% formaldehyde to improve the efficiency of cross-linking. As a result, we found that the terminus is markedly enriched by 3F-ZauP (Fig. 5D). This is not due to an artifact caused by a higher dose of formaldehyde, because ZauP-ChIP recovered only background levels of the origin DNA. Besides, neither the terminus nor the origin was enriched in control ChIP analyses using the wild-type NA1000 strain. These findings suggested that ZauP is able to recruit ZapT in complex with the terminus DNA.

### A fluorescent protein bearing the C-terminal sensor domain of ZapT coincides with ZauP.

**FIG 6:**
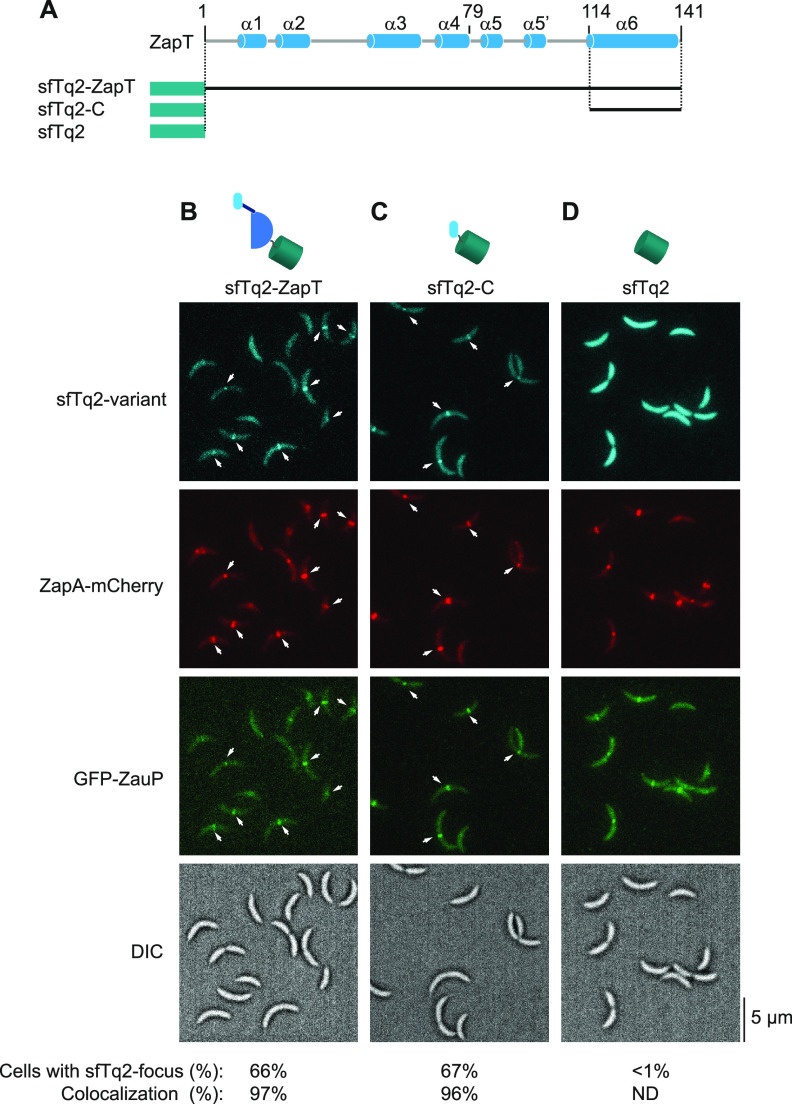
C-terminal sensory domain of ZapT is sufficient for association with the divisome *in vivo*. (A) sfTq2 variants. sfTq2 grafted to the N terminus of full-length ZapT (sfTq2-ZapT), the ZapT C terminus spanning from Gly113 to Gly141 (sfTq2-C), or none (sfTq2) are shown schematically. (B to D) Localization of sfTq2-ZapT variants. SHQ230 (NA1000 *gfp-zauP zapA-mCherry*) cells harboring pBXMCS2sfTq2ZapT (sfTq2-ZapT) (B), pBXMCS2-sfTq2-ZapTG113-G141 (sfTq2-C) (C), or pBXMCS2sfTq2 (sfTq2) (D) were grown exponentially in PYE medium at 30°C and subjected to fluorescence microscopy. Representative images of DIC and fluorescence microscopy are shown (with a scale bar). Arrows indicate the positions of discrete sfTq2 foci. A total of 100 cells were picked randomly for each experiment to determine the percentage of cells with a sfTq2 focus. Of these sfTq2 focus-positive cells, the fractions of cells in which the GFP-ZauP, ZapA-mCherry, and sfTq2 foci resided within a ≤5 pixel distance are shown as colocalization (%). ND, not determined.

To corroborate our idea that the C terminus of ZapT mediates its localization to the Z-ring *in vivo*, we investigated whether a fluorescent protein grafted onto the C-terminal domain of ZapT would be recruited to ZauP in living cells (Fig. 6). To monitor the position of ZauP in a cell, we generated a strain (SHQ230) expressing an N-terminal GFP fusion of ZauP from the native *zauP*-*zapA* operon of the chromosome. In addition, the *zapA* gene of SHQ230 was replaced by a gene encoding a C-terminal mCherry fusion of ZapA, which marks the position of the Z-ring (24, 26). Previously, ZauP was reported to colocalize with ZapA and the Z-ring (40). When SHQ230 was transformed with a low-copy-number plasmid with a superfolder mTurquoise 2 fluorescent protein grafted onto the N terminus of full-length ZapT (sfTq2-ZapT), approximately 66% of the cells had a discrete sfTq2 focus (Fig. 6A and B). In this assay, production of sfTq2-ZapT relied on leaky expression from the xylose promoter in the absence of the inducer xylose, which might result in moderately compromised formation of the focus. Of these cells with an sfTq2 focus, 97% of the transformed cells contained a discrete sfTq2-ZapT focus at a position in close proximity to the GFP-ZauP and ZapA-mCherry foci (Fig. 6B). Moreover, demographic representation of sfTq2-ZapT largely coincided with that of GFP-ZauP and ZapA-mCherry (Fig. S4). These observations are consistent with the idea that ZapT, ZauP, and ZapA form a ternary complex *in vivo*. Notably, when a plasmid expressing sfTq2 grafted onto the N terminus of the truncated ZapT bearing its C-terminal region between Gly113 and Gly141 (sfTq2-C) alone was transferred to SHQ230, the resultant strain produced cells with a discrete sfTq2-C focus that colocalized with GFP-ZauP and ZapA-mCherry foci with a frequency comparable to that of the sfTq2-ZapT strain (Fig. 6A and C). Consistently, demographic representation of sfTq2-C was similar to that of sfTq2-ZapT (Fig. S4). As a control, expression of sfTq2 alone yielded cells with dispersed sfTq2 signals, affecting the formation of neither GFP-ZauP nor ZapA-mCherry foci (Fig. 6A and D and Fig. S4). Together, these results argue that the ZapT C terminus includes a functional motif that interacts with ZauP to form a cluster *in vivo*.

10.1128/mBio.02196-20.4 FIG S4 sfTq2-ZapT colocalization with ZauP and ZapA. Related to Fig. 6, the localization of sfTq2 variants, ZapA-mCherry and GFP-ZauP, were analyzed demographically. Demographic representations were generated using Oufti software. Download FIG S4, TIF file, 2.5 MB. Copyright © 2021 Ozaki et al. 2021 Ozaki et al. This content is distributed under the terms of the Creative Commons Attribution 4.0 International license.

**FIG 7:**
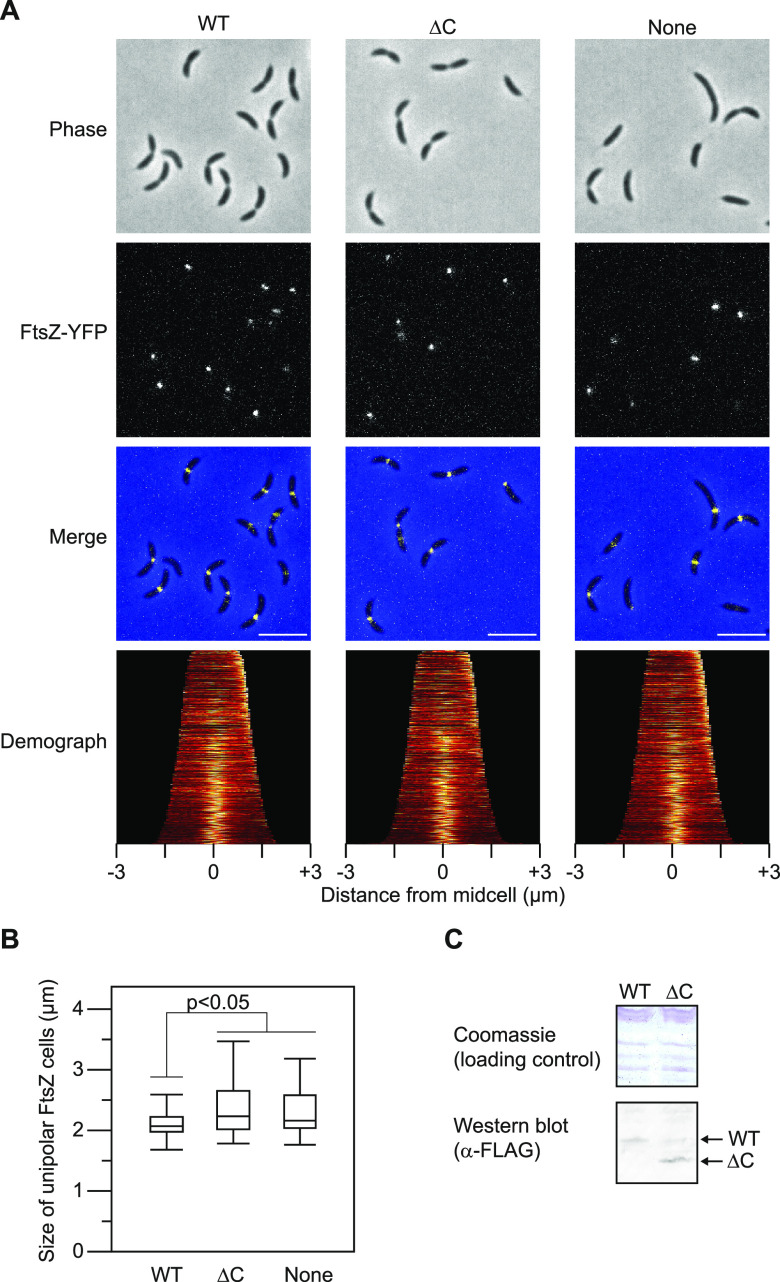
C terminus of ZapT is important for FtsZ positioning. SHQ136 (Δ*zapT vanA*::*ftsZ-yfp*) cells harboring pQF::zapT-3F (WT), pQF::zapTΔC-3F (ΔC), or the empty vector pQF (None) were grown exponentially in PYE medium. After the induction of FtsZ-YFP by treatment with 1 mM vanillate for 1 h, phase-contrast and fluorescent images were taken using fluorescence microscopy. (A) Representative images and demographs generated using Oufti software are shown. For cells with a unipolar FtsZ-YFP focus, the FtsZ-marked cell pole was defined as a new pole. (B) Size distribution of cells with a unipolar FtsZ focus. Size distributions of cells with a unipolar FtsZ focus are shown as a box plot. The *P* value was calculated using the Mann-Whitney-Wilcoxon test. (C) Protein levels were determined using Western blotting with an anti-Flag antibody (1:500).

Finally, to further gain physiological insight into the role of the ZapT-ZauP interaction, we analyzed the localization of the Z-ring using a C. crescentus strain expressing an FtsZ-yellow fluorescent protein (YFP) fusion ectopically from the vanillate-dependent promoter (15). In wild-type cells, a discrete YFP focus is formed at the new cell pole in shorter G_1_ cells and relocates to the midcell region as the cell grows. We previously reported that the timing of FtsZ relocation from the cell pole to midcell is slightly delayed in the Δ*zapT* mutant strain (24). Consequently, Δ*zapT* cells with unipolar FtsZ focus are more elongated than wild-type cells with unipolar FtsZ. Consistent with this, when the size distributions of those unipolar FtsZ cells were analyzed, Δ*zapT* mutant cells bearing the plasmid with wild-type *zapT* were shorter than those with the empty vector (Fig. 7A and B). Strikingly, introduction of the plasmid with the *zapTΔC* allele failed to suppress the observed phenotype of the Δ*zapT* mutant, although expression of ZapT and ZapTΔC was comparable (Fig. 7B and C). Signal intensities of FtsZ-YFP foci appeared unaffected in these strains (Fig. 7A). Thus, these observations argued that ZauP interaction with the C terminus of ZapT is important for the timely formation of the Z-ring at midcell *in vivo*.

## DISCUSSION

**FIG 8:**
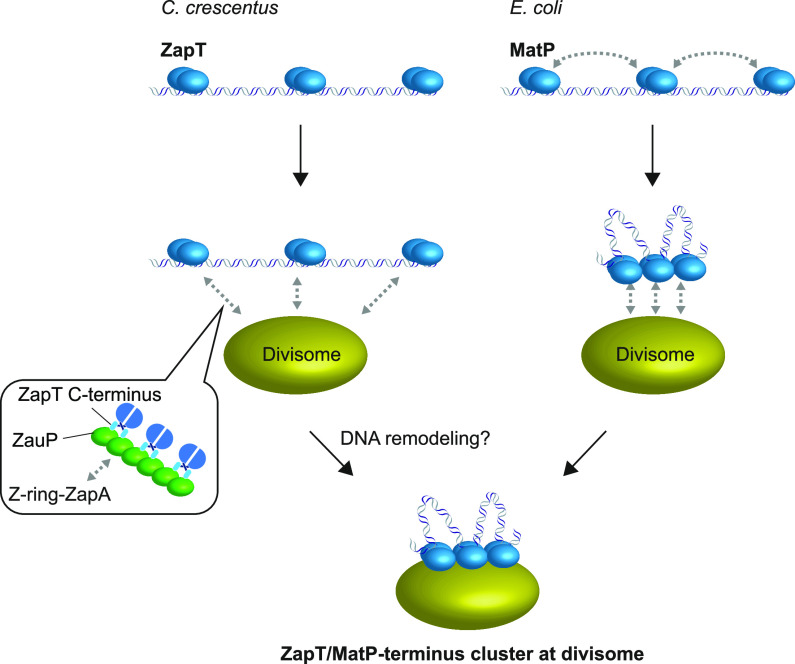
Two distinct processes of terminus-divisome nucleoprotein complex assembly. Individual ZapT-DNA complexes form a cluster in a divisome-dependent manner. In contrast, MatP-DNA complexes can self-organize into a cluster that associates with the divisome. In either process, terminus-divisome nucleoprotein complexes are ultimately formed to spatially coordinate between chromosome positioning and cell division.

In C. crescentus, the replication terminus and divisome are spatially coordinated, localizing in proximity to each other throughout most of the cell cycle. ZapT acts as a terminus-binding protein, physically linking the terminus and divisome. However, the functions and molecular mechanisms of this linkage remain obscure. In this study, we found that ZapT and divisome components ZauP and ZapA interact directly, presumably in that order, to form ternary complexes. Focusing on the ZapT-ZauP interaction, we found that ZapT forms a stable dimer and that multiple ZapT dimers assemble on ZauP oligomer(s). At the early stage of the cell cycle, ZauP is recruited to the Z-ring in a ZapA-dependent manner (40). Therefore, it is reasonable to assume that multiple ZapT molecules are recruited to the early divisome through the interaction with ZauP. Consistent with this idea, we observed that subcellular localization of ZapT depends on ZauP and ZapA and parallels the localization of those two factors. Notably, in mutant cells lacking ZauP or ZapA, ZapT retained a specific affinity for the terminus DNA. Thus, these observations suggested that staged assembly of the protein complexes underlies the physical linkage between divisome and the terminus, i.e., ZapT initially binds to the terminus DNA independently of ZauP and ZapA and subsequently forms a nucleoprotein cluster on the early divisome in a manner dependent on ZauP and ZapA (Fig. 8). We infer that this cluster helps to organize ZapT-bound DNA loci into a compact structure. To the best of our knowledge, this is the first evidence that chromosome organization is driven by the early divisome. Notably, ZapT and ZauP homologs are widespread in diverse Gram-negative pathogens, including the alphaproteobacterium Brucella abortus, the betaproteobacterium Paraburkholderia rhizoxinica, and the gammaproteobacterium Pseudomonas aeruginosa. Sequence alignment revealed that the ZapT C-terminal domain contains several leucine residues that are highly conserved among ZapT homologs (see Fig. S1 in the supplemental material), suggesting that a hydrophobic interaction underlies the interaction between ZapT and ZauP homologs. Hence, our findings may reveal a general mechanism by which the chromosome terminus is coordinated with the divisome in diverse bacterial species.

The interaction between the C terminus of ZapT and ZauP provides mechanistic insight into the organization of the terminus DNA in C. crescentus. ZapT belongs to the MerR family proteins, which contain an N-terminal DNA-binding domain and a C-terminal sensor domain to which cognate effector molecules bind to remodel the DNA structure (41, 42, 44) (Fig. S1). The effectors include heavy metals, chemicals, and oligopeptides. Recent structural and biochemical studies suggest that a MerR family transcriptional regulator forms a homodimer to recognize its cognate DNA within the target promoter regardless of whether the specific effector is present and that binding of the effector induces a scissor-like movement of the dimerization helices. Consequently, the altered domain constellation enables the dimer to introduce a distortion in the DNA structure; this, in turn, promotes transcriptional initiation by RNA polymerase (45, 46). By analogy, a similar conformational change could operate through binding of the ZapT C terminus to ZauP, leading to remodeling of ZapT-bound DNA in the terminus region. Chromosome contact mapping of wild-type C. crescentus revealed that the chromosome comprises multiple, largely independent spatial domains (47, 48). Of these, the largest one is located at 1.6 to 1.9 kb, which overlaps the region to which ZapT binds preferentially. Therefore, it is plausible that ZapT, together with the divisome, plays a part in construction of the chromosome interaction domains at the terminus. The observation that DNA recovery in the ZapT-ChIP assays is moderately elevated in the absence of ZapA or ZauP also implies that the divisome could function in regulatory tuning of ZapT distribution along the DNA.

The function of ZapT contrasts with that of the E. coli terminus-binding protein MatP, which organizes the chromosome structure of the replication terminus independently of the divisome (Fig. 8). Biochemical and structural studies suggest that DNA-bound MatP dimers interact with each other to self-organize a nucleoprotein cluster, shaping the chromosome terminus region into a condensed structure (33). Although MatP interacts with the Z-ring through interaction with ZapB, mutant cells lacking ZapB are still able to form a discrete focus of MatP-fused fluorescent proteins and largely maintain the same chromosome structure at the replication terminus as wild-type cells (29, 31). These observations contrast with the ZauP-dependent clustering of the ZapT-DNA complexes in C. crescentus. Given that MatP and ZapB homologs are conserved only in enteric-related species of gammaproteobacteria, the ability of a terminus recognition protein to self-organize the nucleoprotein cluster might have been acquired late in gammaproteobacterial evolution. In this context, we infer that self-organized clustering of terminus recognition proteins generates multiple contact points by which the overall affinity to the divisome is markedly reinforced, likely through a linkage effect (49).

Faithful segregation of replicated sister chromosomes relies on accurate spatial control of specific chromosome loci with which dedicated proteins form clusters of nucleoprotein complexes. In C. crescentus, ParB binds the origin-proximal *parS* site to form a partition complex that is essential for polar sequestration of the origin (13, 14, 16, 17). The subcellular localization of the origin is also ensured by structural maintenance of chromosome (SMC) complexes (50, 51). Together with accessory proteins ScpA and ScpB, SMC forms a ring-shaped complex within which DNA strands are topologically entrapped (48). Loading of SMC onto the chromosome is stimulated at *parS*, thereby assisting in chromosome organization near the origin. In E. coli, the SMC family DNA-binding protein MukB also plays a crucial role in chromosome segregation (34, 52–54). As with SMC of C. crescentus, MukB localizes primarily in the vicinity of the origin and organizes the chromosome into a compact structure (55, 56). In addition, E. coli has coopted multiple DNA-associating proteins to spatially control the nascently replicated DNA strands. These include the hemimethylated DNA-binding protein SeqA and the DNA polymerase III clamp subunit-interacting protein CrfC. Both proteins help colocalize the newly synthesized DNA strands at midcell, thereby ensuring chromosome segregation (55, 57–59). Thus, our finding that the divisome can control organization of the ZapT-terminus DNA complexes reveals another layer of regulation of chromosome dynamics in coordination with cell division.

## MATERIALS AND METHODS

### Bacterial strains and DNA.

The strains, plasmids, and primers used in this study are listed in Tables 1, 2, and 3, respectively. *Caulobacter* strains were grown at 30°C in peptone-yeast extract (PYE) supplemented with appropriate antibiotics, as described previously (60, 61). When necessary, cumate (1 μM) or vanillate (1 mM) was added to the culture medium as indicated. Detailed procedures for construction of the strains and plasmids are described in the supplemental material (Text S1).

10.1128/mBio.02196-20.5 TEXT S1 Supplemental methods. Download Text S1, DOCX file, 0.02 MB. Copyright © 2021 Ozaki et al. 2021 Ozaki et al. This content is distributed under the terms of the Creative Commons Attribution 4.0 International license.

**TABLE 1:** Strains used in this study

Strain	Genotype	Reference or source
Caulobacter crescentus		
NA1000	Wild-type Caulobacter crescentus strain	65
SHQ10	NA1000 *CCNA_01434*(*zapT*)*-3F*	24
SHQ48	NA1000 Δ*zapT*	24
SHQ56	NA1000 *zapA-mCherry*	24
SHQ68	NA1000 Δ*CCNA_03356* (*zapA*)	24
SHQ69	NA1000 Δ*CCNA_03357* (*zauP*)	24
SHQ143	NA1000 *zapT*::*mNeonGreen*	24
SHQ153	SHQ68 *zapT*::*mNeonGreen*	This study
SHQ154	SHQ69 *zapT*::*mNeonGreen*	This study
SHQ176	NA1000 pQF::*zapT-3F*	24
SHQ197	SHQ68 Δ*zapT*	This study
SHQ198	SHQ69 Δ*zapT*	This study
SHQ230	SHQ56 *GFP-zauP*	This study
SHQ236	SHQ230 pBXMCS2sfTq2ZapT	This study
SHQ237	SHQ230 pBXMCS2sfTq2G113-G141	This study
SHQ238	SHQ230 pBXMCS2sfTq2	This study
SHQ247	NA1000 *3xFLAG-zauP*; replica of UJ9492	24
Escherichia coli		
DH5α	General cloning strain	Invitrogen
Rosetta 2(DE3)	Strain for overproduction of recombinant protein	Novagen

**TABLE 2:** Plasmids used in this study

Plasmid	Description	Reference or source
mNG-sfTq2	Plasmid carrying the mNeonGreen and superfolder mTurquoise 2 (sfTq2) genes	Addgene
pBXMCS-2	Low-copy-no. kanamycin-resistant vector with the xylose-dependent promoter	66
pBXMCS2sfTq2	pBXMCS-2 derivative with sfTq2	This study
pBXMCS2sfTq2G113-G141	pBXMCS-2 derivative with sfTq2-C	This study
pBXMCS2sfTq2ZapT	pBXMCS-2 derivative with sfTq2-ZapT	This study
pET21a01434_3F6H	pET21a derivative for purification of ZapT-His	24
pET21a01434_3F6H_113_del	pET21a01434_3F6H derivative for purification of ZapTΔC-His	This study
pET28ahisSUMO01434	pET28a derivative for purification of N-terminal His-SUMO-tagged ZapT	This study
pEThisMBPzapA	pET28a derivative for purification of N-terminal His-MBP-tagged ZapA	This study
pEThisSUMOzauP	pET28a derivative for purification of N-terminal His-SUMO-tagged ZauP	This study
pNPTS01434-CKO	Suicide vector for introduction of an in-frame deletion of *zapT*	24
pNPTS01434-mNG	pNPTS138 derivative with *zapT-mNeonGreen*	24
pNPTS-GFP-zauP	Suicide vector for grafting GFPmut3 onto the N terminus of ZauP	This study
pQF	Low-copy-no. tetracycline-resistant vector with the cumate-dependent promoter	67
pQF::zapT-3F	pQF derivative with *zapT-3F*	24
pQF::zapTmNG	pQF derivative with *zapT-mNeonGreen*	This study
pQF::zapTΔC-3F	pQF::zapT-3F derivative with the *zapTΔC*(*2-113aa*) allele	This study

### Recombinant proteins.

Recombinant proteins were expressed in E. coli and purified as described in the supplemental material (Text S1).

### Size exclusion chromatography.

The size exclusion chromatography assay was performed essentially as described previously (57, 62). Briefly, proteins were loaded onto a Superdex 200 PC3.2/30 column (2.4-ml column volume) equilibrated with SEC buffer (25 mM Tris-HCl [pH 7.5], 300 mM sodium chloride, and 20% sucrose) and fractionated at a flow rate of 20 μl/min, followed by SDS–15% PAGE and Coomassie brilliant blue staining. When a Superdex 75 HR 10/30 column was used, proteins were separated at a flow rate of 0.2 ml/min.

### ChIP sequencing and ChIP-qPCR.

ChIP was performed as described previously (24). Briefly, exponentially growing cells (200 ml in PYE) were fixed for 10 min using 1% formaldehyde solution, washed thoroughly, resuspended in buffer, and lysed through two passages in a French press. After DNA shearing with sonication, the cell debris was removed by ultracentrifugation, and the cleared cell lysate was incubated with anti-FLAG M2 magnetic beads (Wako or Sigma). After the beads were washed, the bound materials were incubated at 65°C overnight to reverse cross-linking. The resultant DNA samples were purified using a DNA cleanup kit (Zymo Research). When SHQ247 (3F-ZauP) cells (100 ml in PYE) were analyzed, cross-linking was carried out for 5 min in 3.6% formaldehyde instead of 1% formaldehyde.

For deep sequencing, samples were indexed using a NEBNext Ultrall DNA library prep kit and analyzed on an Illumina HiSeq 2500 instrument (single-end).

For qPCR, samples were analyzed by a standard percent input method using TB green premix ExTaqII and Thermal Cycler Dice TP800 (TaKaRa). Locus-specific primers are listed in Table 3.

**TABLE 3:** Oligonucleotides used in this study

Name	Sequence (5′–3′)
9	GTCGGAAAAACTTCTCGCGG
10	ATCGGGCTTTCGATCTGCTT
11	GCCTTCCCACATGGGGTT
12	CTGTCGTGTCTCAGGACGTT
148	GCCGACAGGGCGCTCTTTGCCCCGC
149	GGCGGCGGCGACTACAAAGACCATG
253	TGGGAGCTCGAAGGAGATATACCATGGCGAAGGGGCCAAACGCCTTCCG
254	GACAAGCTTCAACCGCGCGCCAAAAGTCCGTCGA
273	GCCACCAATCTGTTCGCGGTGAGCC
274	ATGGCGAAGGGGCCAAACGCCTTCC
459	AGGAAGCTTCCATATGGCGAAGGGGCCAAACGCCTTCCG
460	GCCGAATTCCGACGCGGAAGGAGCGCCCTTAT
637	CCGCATATGAGTAAAGGAGAAGAACTTTTCA
638	TGCGGAAGGCGTTTGGCCCCTTCGCGGTACCCTTGTACAGCTCGTCCATGCCGAGA
639	TCTCGGCATGGACGAGCTGTACAAGGGTACCGCGAAGGGGCCAAACGCCTTCCGCA
640	ACAGAATTCAACCGCGCGCCAAAAGTCCGTCG
641	CGCGGTACCCTTGTACAGCTCGTCC
642	CGCGGTACCGGCGAGGAGACGCGGGACCGACTGG
649	CTGACGCGTGGAACTGGCGCTAG
650	AATTCTAGCGCCAGTTCCACGCGTCAGGTAC
683	GCCGGAATTGACGATCCAGT
684	ACCTGTCGTACTTCGTTCGC
687	ATGGACGTTGGCGTAAGAGG
688	CACGGCAAGCCGTTGATTTAT
694	TACTGGGAAGAGCGGTGGAT
695	AATGATCGACATCCGAGGCG
zapA-zapB_down-fwd-BamHI	AGCGGATCCTTAGTCATCAAGAATAAAAGCAAC
zapA-zapB_up_rev-EcoRI	GGCGAATTCCTTGCTGGTGAAGATGCCGGTG
GFP-zapB-up-rev	TGAAAAGTTCTTCTCCTTTACTCATCGGCGGAAATCCATCATGCGACGCA
GFP-zapB-mid-fwd	TGCGTCGCATGATGGATTTCCGCCGATGAGTAAAGGAGAAGAACTTTTCA
GFP-zapB-mid-rev	GTACTGTCGGCCGGGATCATGGTACCGCCAGAACCAGCAGCGGAGCCAGC
GFP-zapB-down-fwd	CTCCGCTGCTGGTTCTGGCGGTACCATGATCCCGGCCGACAGTACGGCCC
NhisMBP-zapA-fwd-BamHI	GCCGGATCCATGGCTCAGGTGACCATCCA
NhisMBP-zapA-rev-HindIII	CACAAGCTTAGTCATCAAGAATAAAAGCAAC
Nhis-SUMO-zapB-P1-NcoI	ATACCATGGGTCATCACCATCATCA
Nhis-SUMO-zapB-P2	GGGCCGTACTGTCGGCCGGGATCATGCCACCAATCTGTTCGCGGTGAGCC
Nhis-SUMO-zapB-P3	GGCTCACCGCGAACAGATTGGTGGCATGATCCCGGCCGACAGTACGGCCC
Nhis-SUMO-zapB-P4-SacI	CCAGAGCTCAGGCCTCCTCGGAGTCTTCGAAC

### Microscopy.

Differential interference contrast (DIC), phase-contrast, and fluorescence microscopy analyses were performed using a Nikon Eclipse 80i microscope equipped with an X-Cite TURBO multiwavelength LED illumination system and an Andor Zyla 4.2 sCMOS camera, as described previously (24). Quantitative image analyses were performed using the Oufti and MicrobeJ software packages (63, 64).

### Western blotting.

The Western blot assay was performed as described previously (24). The anti-mNeonGreen and anti-Flag antibodies were purchased from Chromotek and Thermo, respectively.
