# Supplementary figures and images for: Z-Ring-Associated Proteins Regulate Clustering of the Replication Terminus-Binding Protein ZapT in Caulobacter crescentus
Source: mBio. 2021 Jan 26;12(1):e02196-20. doi: 10.1128/mBio.02196-20 (PMC7858052; doi:10.1128/mBio.02196-20)

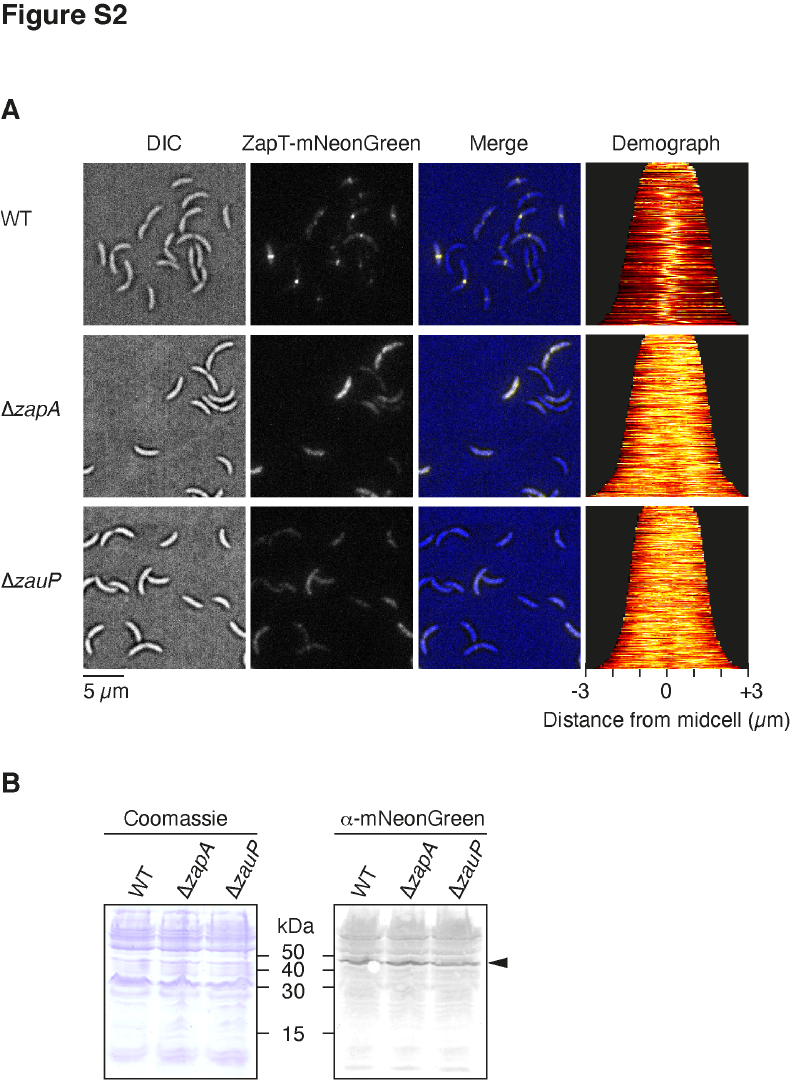

Supplement: FIG S2 [file mBio.02196-20-sf002.tif]

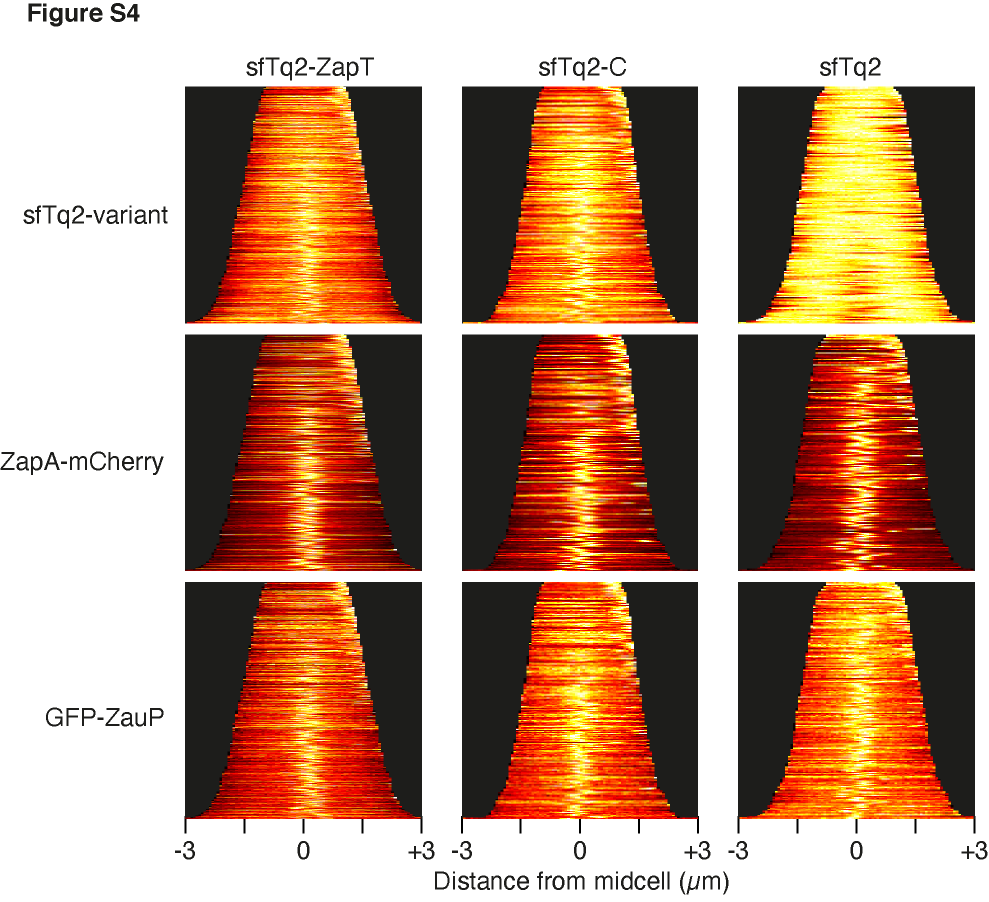

Supplement: FIG S4 [file mBio.02196-20-sf004.tif]
