# Supplementary material for: Z-Ring-Associated Proteins Regulate Clustering of the Replication Terminus-Binding Protein ZapT in Caulobacter crescentus
Source: mBio. 2021 Jan 26;12(1):e02196-20. doi: 10.1128/mBio.02196-20 (PMC7858052; doi:10.1128/mBio.02196-20)
Supplement: TEXT S1 [file mBio.02196-20-s0001.docx]

**Supplemental material**

**Bacterial strains**

The SHQ68 derivative strains SHQ153 and SHQ197 were generated by double homologous recombination using pNPTS01434-CKO and pNPTS01434-mNG, respectively. SHQ154 and SHQ198 were generated similarly using SHQ69 instead of SHQ68 as a parental strain. SHQ230 was generated by double homologous recombination using SHQ56 as a parental strain and pNPTS-GFPzapB. Plasmid DNAs were introduced into parental *C. crescentus* strains by electroporation.

**Plasmid construction**

To generate pBXMCS2sfTq2ZapT, a 1.2-kb insert DNA was generated by overlap extension PCR. First, a 751-bp DNA was amplified by PCR using mNG-sfTq2 and primers 637/638. Second, a 461-bp DNA was amplified by PCR using NA1000 and primers 639/640. These fragments were combined by overlap extension PCR with primers 637/640. After digestion with *Nde*I and *Eco*RI, the products were ligated to the *Nde*I–*Eco*RI fragment of pNPTS138.

To generate pBXMCS2sfTq2G113-G141, a 6.1-kb DNA was amplified by PCR using pBXMCS2sfTq2ZapT and primers 641/642. After digestion with *Kpn*I, the product was self-ligated.

To generate pBXMCS2sfTq2, the *zapT*-encoding region was digested with *Kpn*I and *Eco*RI, followed by ligation to an oligonucleotide cassette consisting of primers 649/650.

To generate pET21a01434_3F6H_113_del, a 5.8-kb DNA was amplified by PCR using pET21a01434_3F6H and primers 148/149, followed by self-ligation.

To generate pET28ahisSUMOzauP, a 0.7-kb insert DNA was generated by overlap extension PCR. First, a 351-bp DNA was amplified by PCR using primers Nhis-SUMO-zapB-P1-NcoI/Nhis-SUMO-zapB-P2, with vector hisSUMO as the template. Second, a 416-bp DNA was amplified by PCR using primers Nhis-SUMO-zapB-P3/Nhis-SUMO-zapB-P4-SacI, with NA1000 genomic DNA as the template. These fragments were combined by overlap extension PCR with primers Nhis-SUMO-zapB-P1-NcoI/Nhis-SUMO-zapB-P4-SacI. After digestion with *Nco*I and *Sac*I, the products were ligated to the *Nco*I–*Sac*I fragment of pET28a.

To generate pET28ahisSUMO01434, a 457-bp DNA containing the CCNA_01434 ORF was amplified by PCR using primers 253/254, with NA1000 genomic DNA as the template. The product was cut with *Sac*I and *Hin*dIII and ligated to the *Sac*I–*Hin*dIII fragment of pET28ahisSUMOzapB. The resultant plasmid and primers 273/274 were used for PCR to amplify a 6.0-kb DNA. The products were self-ligated, yielding pET28ahisSUMO01434

To generate pEThisMBPzapA, a 366-bp DNA was amplified by PCR using NA1000 genomic DNA and primers NhisMBP-zapA-fwd-BamHI/NhisMBP-zapA-rev-HindIII. After digestion with *Bam*HI and *Hin*dIII, the product was ligated to the *Bam*HI–*Hin*dIII fragment of pEThisMBP.

To generate pNPTS-GFPzauP, a 2.0-kb insert DNA was generated by overlap extension PCR. First, a 0.56-kb DNA was amplified by PCR using NA1000 genomic DNA and primers zapA-zapB_up_rev-EcoRI/GFP-zapB-up-rev. Second, a 0.79-kb DNA was amplified by PCR using GFPmut3 vector and primers GFP-zapB-mid-fwd/GFP-zapB-mid-rev. Third, a 0.77-kb DNA was amplified by PCR using NA1000 genomic DNA and primers GFP-zapB-down-fwd/zapA-zapB_down-fwd-BamHI. These products were combined by overlap extension PCR, yielding a 2.0-kb insert DNA. After digestion with *Eco*RI and *Bam*HI, the products were ligated to the *Eco*RI–*Bam*HI fragment of pNPTS138.

To generate pQF::zapTmNG, a 1203-bp DNA was amplified by PCR using NA1000 genomic DNA and primers 459/460. After digestion with *Hin*dIII and *Eco*RI, the insert was ligated to the *Hin*dIII–*Eco*RI fragment of pQF.

To generate pQF::zapT∆C-3F, a 7.9-kb DNA was amplified by PCR using pQF::zapT-3F and primers 148/149, followed by self-ligation.

**Proteins**

ZapT-His and ZapT∆C-His were purified essentially as described previously. Briefly, Rosetta™ 2(DE3) (Novagen) harboring pET21a01434_3F6H was grown exponentially in LB medium supplemented with ampicillin and chloramphenicol, and expression of His-tagged proteins was induced at 37˚C for 3 h by addition of 1 mM isopropyl-β-D-thiogalactoside. The induced cells were harvested by centrifugation and resuspended in buffer A (25 mM Tris-HCl [pH 7.5], 300 mM sodium chloride, and 5% glycerol) supplemented with 5 mM imidazole, 0.1% Triton X-100, and 0.2 mg/mL lysozyme. The cell suspension was incubated on ice for 30 min, frozen in liquid nitrogen, and thawed to lyse the cells. After ultracentrifugation (48000 rpm, 20 min), the supernatant was loaded onto a Ni Sepharose 6 Fast Flow column (0.5 mL) equilibrated with buffer A containing 5 mM imidazole. After washing with buffer A containing 40 mM imidazole, ZapT-His proteins retained on the column were eluted in buffer A containing 500 mM imidazole. ZapT-His was further purified using HiTrap heparin (1 mL): ZapT-His was loaded in buffer B (25 mM Tris-HCl [pH 7.5], and 5% glycerol) containing 100 mM sodium chloride, washed using a linear gradient (200–500 mM sodium chloride) in buffer B. ZapT-His proteins were eluted at 1000 mM sodium chloride.

To purify native ZapT, Rosetta™ 2(DE3) cells harboring pET28ahisSUMO01434 were grown exponentially in LB medium (1.5 L) supplemented with kanamycin and chloramphenicol, and expression of N-terminal His-SUMO-tagged ZapT was induced at 37˚C for 1 h by addition of 1 mM isopropyl-β-D-thiogalactoside. The induced cells were harvested by centrifugation and resuspended in 17 mL Ni-A buffer (50 mM monosodium phosphate [pH 7.5], 200 mM sodium chloride, 10 mM imidazole, 50 mM l-glutamic acid, and 50 mM l-arginine) containing 0.5% dodecyl maltoside and 0.2 mg/mL lysozyme. The cell suspension was incubated on ice for 30 min, frozen in liquid nitrogen, and thawed to lyse the cells. After ultracentrifugation (48000 rpm, 20 min), the supernatant was loaded onto a Ni Sepharose 6 Fast Flow column (1 mL) equilibrated with Ni-A buffer containing 0.05% DDM and washed in 15 mL of Ni-A buffer containing 0.05% DDM (15 mL). After additional washing in 15 mL of Ni-A’ buffer (25 mM Tris-HCl [pH 7.5], 5% glycerol, 200 mM sodium chloride, 30 mM imidazole, 50 mM l-glutamic acid, and 50 mM l-arginine), proteins were eluted in Ni-E buffer (25 mM Tris-HCl [pH 7.5], 5% glycerol, 200 mM sodium chloride, 500 mM imidazole, 50 mM l-glutamic acid, and 50 mM l-arginine). After dialysis against buffer Ni-D buffer (25 mM Tris-HCl [pH 7.5], 5% glycerol, 200 mM sodium chloride, 50 mM l-glutamic acid, and 50 mM l-arginine), the sample (2.3 mg/mL, 7mL) was mixed with SUMO protease His-Ulp1 (16 µg) and incubated with gentle agitation for > 12h in a cold room to cleave the His-SUMO tag. His-tagged proteins were removed by passage through a Ni Sepharose 6 Fast Flow column, yielding native ZapT (11 mL). A portion (5 mL) was mixed with the same volume of buffer Ni-D’ (25 mM Tris-HCl [pH 7.5], 5% glycerol, 50 mM glutamic acid, 50 mM arginine) and loaded onto HiTrap heparin equilibrated with buffer Ni-D’ containing 100 mM sodium chloride. Proteins were eluted by a linear gradient (0.1–1 mM sodium chloride) in buffer Ni-D’. Elution peak fractions were pooled.

To purify His-ZapA, Rosetta™ 2(DE3) cells harboring pEThisMBPzapA were grown exponentially in LB medium (1 L) supplemented with kanamycin and chloramphenicol, and His-ZapA was induced at 37˚C for 3 h by addition of 1 mM isopropyl-β-D-thiogalactoside. The induced cells were harvested by centrifugation and resuspended in buffer A supplemented with 0.1% Brij, 5 mM imidazole, and 0.2 mg/mL lysozyme. The cell suspension was incubated on ice for 30 min, frozen in liquid nitrogen, and thawed to lyse the cells. After ultracentrifugation (48000 rpm, 20 min), the supernatant was loaded onto a Ni Sepharose 6 Fast Flow column (1 mL) equilibrated with buffer A containing 5 mM imidazole. After washing with buffer A containing 40 mM imidazole, proteins were eluted in buffer A containing 500 mM imidazole. Elution (3 mL) diluted with 7 mL of buffer B was loaded onto a HiTrapQ column equilibrated with buffer containing 100 mM sodium chloride, and proteins were fractionated using a linear gradient (200–1000 mM sodium chloride) of buffer B. His-ZapA proteins eluted at 200 mM sodium chloride were pooled (1 mg/mL, 1mL).

To purify His-ZauP, Rosetta™ 2(DE3) cells harboring pEThisSUMOzauP were grown exponentially in LB medium (1 L) supplemented with kanamycin and chloramphenicol, and His-ZauP was induced at 37˚C for 3 h by addition of 1 mM isopropyl-β-D-thiogalactoside. Following cell lysis, Ni-affinity chromatography and anion exchange chromatography were carried out as described above for His-ZapA purification. Eluate (3 mL) diluted with 7 mL of buffer A’ (no salt) was loaded onto a HiTrapQ column and proteins were eluted using a linear gradient (200–1000 mM sodium chloride) in buffer A. His-ZauP proteins eluted in 400 mM sodium chloride were pooled (3 mg/mL, 2 mL). Native ZauP was purified using His-Ulp1 essentially as described above for native ZapT purification.

To purify His-Ulp1, Rosetta™ 2(DE3) cells harboring pULP1 (a gift from Dr. Daisuke Takahashi) were grown exponentially in LB medium (0.5 L) supplemented with ampicillin and chloramphenicol, and His-Ulp1 was induced at 16˚C for 20 h by addition of 0.5 mM isopropyl-β-D-thiogalactoside. The induced cells were harvested by centrifugation and resuspended in buffer U1 (50 mM sodium phosphate [pH 8.0], 300 mM sodium chloride, and 10 mM imidazole) containing protease inhibitor cOmplete (Roche), and lysed by sonication. After ultracentrifugation (48000 rpm, 20 min), the supernatant was loaded onto a Ni Sepharose 6 Fast Flow column (1 mL) equilibrated with buffer U1. After a wash with 30 mL of buffer U2 (50 mM sodium phosphate buffer [pH 8.0], 300 mM sodium chloride, and 50 mM imidazole), proteins were eluted in buffer U3 (50 mM sodium phosphate buffer [pH 8.0], 300 mM sodium chloride, and 300 mM imidazole). The peak fraction was concentrated by ultrafiltration (Amicon Ultra MWCO=10K; Millipore) and applied to a Superdex 200 column (24 mL) equilibrated with buffer U4 (50 mM Tris-HCl [pH 8.0], 500 mM sodium chloride, 1 mM DTT, and 1 % glycerol). Peak fractions were mixed with glycerol (final 50%), and aliquots were frozen.
